# Prognostic significance of PNH clones in aplastic anemia treated with immunosuppression or allogeneic HSCT: a 20-year single-center experience

**DOI:** 10.1007/s44313-026-00136-3

**Published:** 2026-04-22

**Authors:** Alfadil Haroon, Hazzaa Alzahrani, Mostafa F. Mohammed Saleh, Ali Aalahmari, Shaykhah Alotaibi, Tusneem Elhassan, Feras Alfraih, Fahad Alsharif, Syed O. Ahmed, Fahad Almohareb, Riad El Fakih, Mahmoud Aljurf

**Affiliations:** 1 Adult Hematology, stem cell transplant and cellular therapy section, Cancer Center of Excellence King Faisal Specialist Hospital and Research Center, PO Box 3354, Riyadh, 11471 Saudi Arabia; 2 Clinical Hematology Unit, Internal Medicine Department, Assiut University Hospital, Assiut, Egypt

**Keywords:** Aplastic anemia, PNH clone, Immunosuppressive therapy, Allogeneic stem cell transplantation, Prognosis, GVHD

## Abstract

**Background:**

Paroxysmal nocturnal hemoglobinuria (PNH) clones are detected in up to 60% of patients with aplastic anemia (AA); however, their prognostic impact remains incompletely defined, particularly in the context of frontline immunosuppressive therapy (IST) or allogeneic hematopoietic stem cell transplantation (HSCT).

**Methods:**

We retrospectively analyzed 207 patients with AA treated between 2004 and 2024 at a single institution. PNH clones were identified at diagnosis in 64 patients (30.9%). Treatment modalities included IST (*n* = 104) and HSCT (*n* = 103). Clinical outcomes, including overall survival (OS), event-free survival, graft-versus-host disease (GVHD), relapse, and non-relapse mortality, were compared between the PNH-positive and PNH-negative cohorts.

**Results:**

At 5 years, PNH-positive patients treated with IST had significantly improved OS compared with PNH-negative patients (100% vs. 72.4%, *p* = 0.004). In the HSCT group, OS was 100% in PNH-positive patients versus 90% in PNH-negative patients (*p* = 0.09). The incidence of chronic GVHD after HSCT was significantly lower in the PNH-positive group (4% vs. 27%, *p* = 0.01), whereas the rates of acute GVHD, graft failure, and relapse were comparable. Clone size (small vs. large) did not affect survival or GVHD outcomes.

**Conclusions:**

The presence of a PNH clone in AA was associated with superior survival following IST and a lower incidence of chronic GVHD following HSCT. These findings suggest that PNH positivity may serve as a prognostic and immunomodulatory biomarker in AA and support its integration into therapeutic decision-making and risk stratification algorithms.

## Introduction

Aplastic anemia (AA) is a rare and life-threatening hematologic disorder characterized by pancytopenia and a hypocellular bone marrow. Most acquired cases are immune mediated, in which autoreactive T lymphocytes suppress hematopoietic stem and progenitor cells. Although immunosuppressive therapy (IST) and hematopoietic stem cell transplantation (HSCT) remain the cornerstones of AA management, treatment outcomes vary considerably, particularly in the presence of clonal hematopoiesis [1].

Paroxysmal nocturnal hemoglobinuria (PNH) is a clonal disorder arising from somatic mutations in the PIG-A gene, leading to deficiency of glycosylphosphatidylinositol (GPI)-anchored proteins on blood cells. Approximately 50–60% of patients with AA harbor PNH clones, which may be small and clinically silent or large and associated with hemolytic manifestations. The presence of PNH clones in AA reflects immune selection pressure, and its clinical relevance has gained increasing attention in recent years [2].

Previous studies have demonstrated that the presence of a PNH clone in AA may predict a better hematologic response to IST and more favorable overall survival (OS) [3, 4]. This observation is thought to reflect the immune escape advantage of GPI-deficient cells in an inflammatory marrow milieu [5]. In contrast, large PNH clones may predispose patients to hemolysis, thrombosis, and long-term clonal evolution, including the development of myeloid malignancies [6]. However, the role of PNH clones in patients undergoing HSCT remains less clear, particularly with respect to transplant-related outcomes such as graft failure (GF), graft-versus-host disease (GVHD), and non-relapse mortality (NRM).

This study aimed to explore the prognostic impact of PNH clone status on survival, relapse, and transplant-related outcomes in patients with AA treated with frontline IST or allogeneic HSCT over a 20-year period at a single center. We further assessed whether clone size modifies outcomes and whether differences in GVHD incidence may support a biologically distinct immune profile in PNH-positive patients.

## Patients and methods

### Patients

Patients included in this retrospective analysis were diagnosed with acquired AA at King Faisal Specialist Hospital and Research Centre between January 2004 and December 2024. A total of 207 consecutive patients who received frontline therapy with either IST or allogeneic HSCT were included. The diagnosis of AA was established according to standard criteria, and disease severity was classified using the Camitta criteria. All patients underwent baseline bone marrow evaluation to exclude hypocellular myelodysplastic syndrome and inherited bone marrow failure syndromes. The study was approved by the Institutional Review Board. Baseline assessment included complete blood counts, biochemical parameters, and flow cytometric evaluation for PNH clones. PNH clones were assessed by multiparameter flow cytometry using fluorescent aerolysin (FLAER)-based assays and/or CD55/CD59 testing according to institutional laboratory standards. PNH clone positivity was defined as ≥ 0.01% GPI-deficient granulocytes or red blood cells. Clone size was further categorized as small (< 10%) or large (≥ 10%).

### Treatment

#### Immunosuppressive therapy (IST)

A total of 104 patients (50.3%) received frontline IST. The majority (77 patients, 74%) were treated with antithymocyte globulin (ATG) (horse or rabbit) in combination with cyclosporine, with or without eltrombopag. The remaining 27 patients (26%) received alternative immunosuppressive regimens, including mycophenolate mofetil, eltrombopag monotherapy, danazol, or corticosteroids.

#### Allogeneic HSCT

Frontline HSCT was performed in 103 patients (49.7%). Most transplants were from matched sibling donors (MSD) (98 patients, 95.1%), whereas 4 patients (3.8%) received haploidentical grafts and 1 patient (0.9%) received a matched unrelated donor (MUD) transplant. Conditioning regimens included fludarabine/cyclophosphamide (Flu/Cy) in 70 patients (68.0%), Flu/Cy/rATG in 16 (15.5%), Flu/Cy/rATG with low-dose total body irradiation (TBI) in 8 (7.8%), and cyclophosphamide/ATG alone in 8 patients (7.8%). Bone marrow was the primary graft source in 90.3% of transplants; peripheral blood stem cells were used in 5.8%, and combined sources in 3.9%. GVHD prophylaxis consisted of cyclosporine with methotrexate (CSA/MTX) in 85 patients (82.5%), tacrolimus with methotrexate in 11 (10.7%), and other combinations in 7 (6.8%).

#### Definitions and endpoints

OS was calculated from the date of first complete remission (CR1) to death from any cause. Event-free survival (EFS) was calculated from CR1 to relapse, GF, or death, whichever occurred first. In the IST cohort, events included relapse, death, or the development of clinical PNH. In the HSCT cohort, events were defined as GF, grade 2–4 acute GVHD, chronic GVHD, or death. Primary GF was defined as failure to achieve an absolute neutrophil count > 0.5 × 10^9^/L for three consecutive days by day + 28. Secondary GF was defined as recurrent neutropenia (< 0.5 × 10^9^/L) after initial engraftment, with loss of donor chimerism or the requirement for stem cell reinfusion. Relapse was defined as recurrence of severe AA (SAA) according to standard severity criteria following an initial response to IST, or the reappearance of transfusion dependence or severe neutropenia. NRM was defined as death in the absence of relapse.

### Statistical analysis

Baseline characteristics were summarized using medians and interquartile ranges (IQRs) or proportions, as appropriate. Comparisons between groups were performed using the chi-square test or Fisher’s exact test for categorical variables. Survival probabilities were estimated using the Kaplan–Meier method and compared using the log-rank test. A two-sided *p* value < 0.05 was considered statistically significant.

## Results

### Baseline characteristics

Among the 207 patients included in the analysis, the median age was 23 years (IQR, 18–31), and 109 patients (52.6%) were male. PNH clones were detected at diagnosis in 64 patients (30.9%), whereas 143 patients (69.1%) were PNH negative. Among PNH-positive patients, 41 had small clones (< 10%) and 23 had large clones (≥ 10%). Baseline demographic and disease characteristics were comparable between the PNH-positive and PNH-negative groups (Table 1).

**Table 1:** Baseline Demographic, Disease, and Treatment Characteristics of the Study Cohort

Variables	N (%)	
**Total number of patients**	207	
**Gender**		
Male	109 (52.6%)	
Female	98 (47.3%)	
**Treatment modality**		
IST	104 (50.3%)	
Allogeneic HSCT	103 (49.5%)	
**Donor type**		
MSD	98 (95.1%)	
Haploidentical	4 (3.8%)	
MUD	1 (0.9%)	
**Stem cell source**		
Bone marrow	93 (90.3%)	
Peripheral blood	6 (5.8%)	
Bone marrow and peripheral blood	4 (3.9%)	
**Conditioning regimen**		
Flu/Cy	70 (68.0%)	
Flu/Cy/ATG	16 (15.5%)	
Flu/Cy/ATG/TBI	8 (7.8%)	
Cy/ATG	8 (7.8%)	
**GVHD prophylaxis**		
CSA/MTX	85 (82.5%)	
FK/MTX	11 (10.7%)	
Others (PTCy/MMF/FK or sirolimus + MMF)	7 (6.8%)	
IST **regimen**: CSA/ATG ± eltrombopag	77 (74%)	
Other IST (mycophenolate mofetil [MMF], eltrombopag monotherapy, danazol)	27 (26%)	
**PNH status**		
PNH negative	143 (69.0%)	
PNH positive	64 (30.9%)	
	Small clone 41 (64%)	Large clone 23 (36%)

*AA* aplastic anemia, *PNH* paroxysmal nocturnal hemoglobinuria, *HSCT* hematopoietic stem cell transplantation, *IST* immunosuppressive therapy, *MSD* matched sibling donor, *MUD* matched unrelated donor, *Flu* fludarabine, *Cy* cyclophosphamide, *ATG* antithymocyte globulin, *TBI* total body irradiation, *CSA* cyclosporine A, *FK* tacrolimus, *MTX* methotrexate, *MMF* mycophenolate mofetil, *PTCy* post-transplant cyclophosphamide

### Survival outcomes

**Fig. 1:**
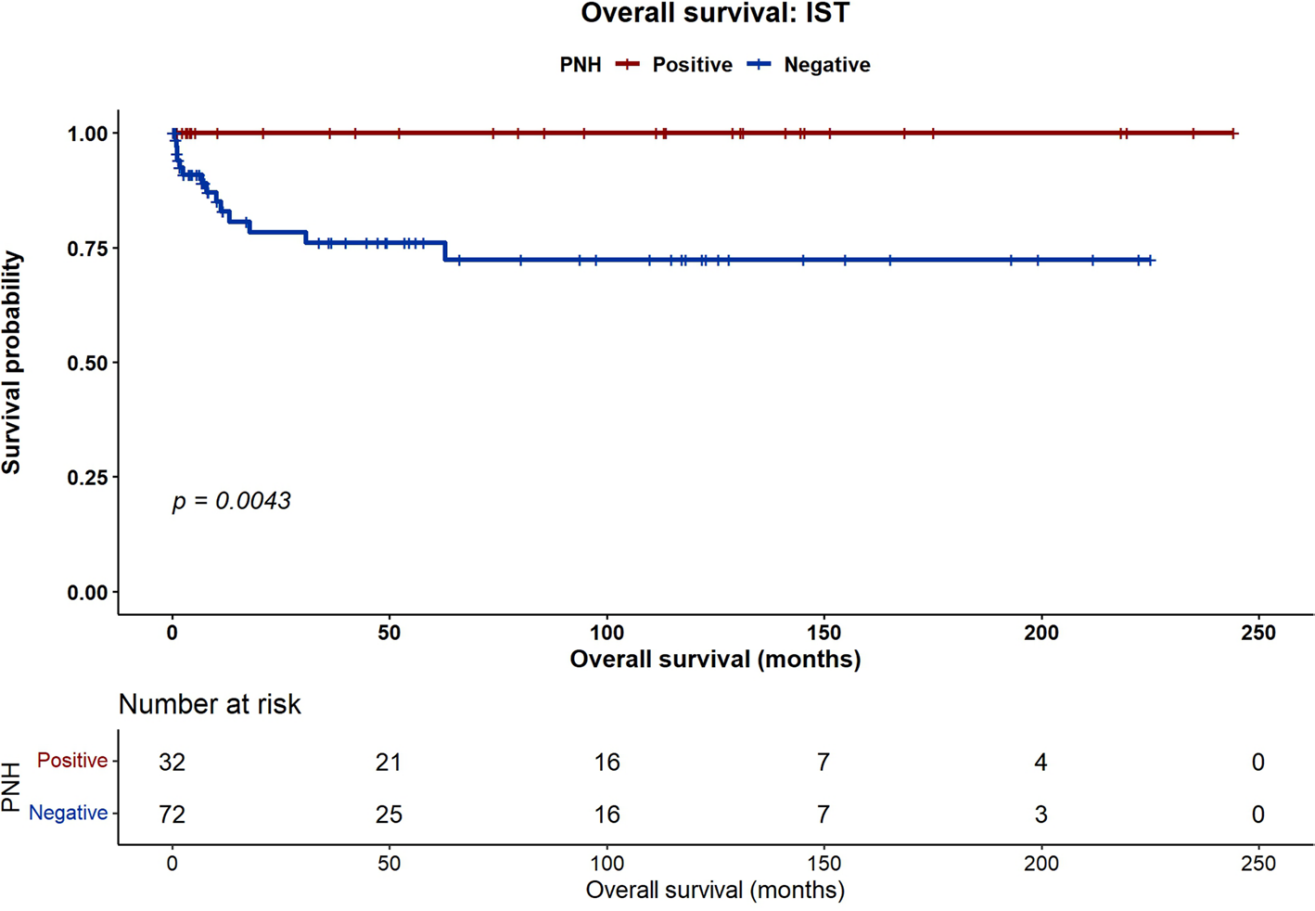
Five-year overall survival in patients receiving first-line immunosuppressive therapy (IST), stratified by PNH clone status

**Fig. 2:**
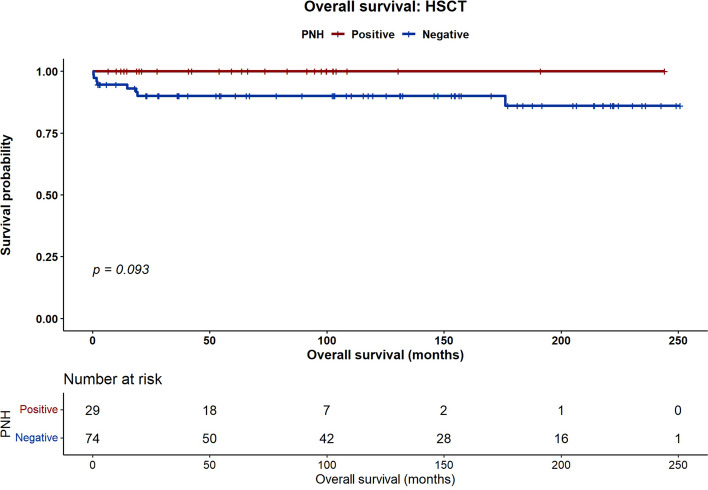
Five-year overall survival following allogeneic HSCT, stratified by PNH clone status

**Fig. 3:**
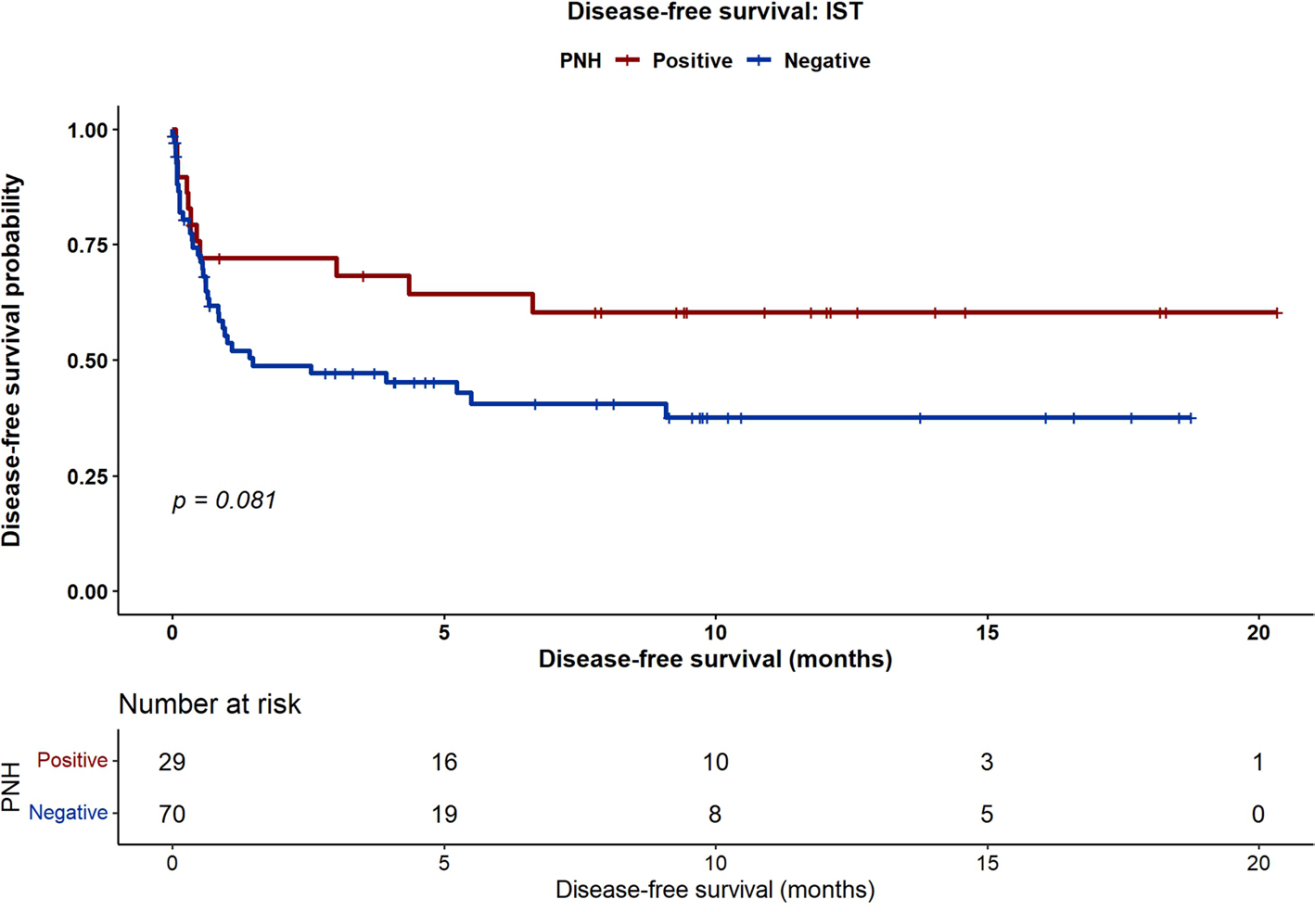
Five-year event-free survival in the IST cohort according to PNH clone status

With a median follow-up of 102 months (95% confidence interval [CI], 90.6–114.3), five-year OS differed according to PNH status. In the IST cohort, five-year OS was significantly higher in PNH-positive patients than in PNH-negative patients (100% vs. 72.4%; *p* = 0.004) (Fig. 1). In the HSCT cohort, five-year OS was 100% in PNH-positive patients and 90% in PNH-negative patients; however, this difference did not reach statistical significance (100% vs. 90%; *p* = 0.09) (Fig. 2). At five years, EFS in the overall cohort was 60% in PNH-positive patients versus 37.6% in PNH-negative patients (*p* = 0.08) (Fig. 3). Although this difference did not reach statistical significance, a numerical trend toward improved outcomes in PNH-positive patients was observed.

### Relapse after IST

Among patients treated with IST, relapse occurred in 36.3% of PNH-positive patients compared with 50% of PNH-negative patients (*p* = 0.2). No statistically significant difference was observed between patients with small and large PNH clones.

### Post-transplant outcomes

In the HSCT cohort, acute GVHD occurred in 10% of PNH-positive patients and 15% of PNH-negative patients (*p* = 0.16). Notably, the incidence of chronic GVHD was significantly lower in PNH-positive patients (4% vs. 27%; *p* = 0.01). GF was observed in 18% of PNH-positive patients and 10% of PNH-negative patients (*p* = 0.5). NRM was 0% in PNH-positive patients compared with 10% in PNH-negative patients (*p* = 0.08); however, this difference did not reach statistical significance. No differences in GVHD incidence, GF, or relapse were observed between patients with small and large PNH clones. Table 2 summarizes the clinical outcomes.

**Table 2:** Comparison of Clinical Outcomes According to PNH Clone Status

Outcome	PNH positive	PNH negative	*P* value
Five-year OS in IST	100%	72.4%	0.004
Five-year OS in HSCT	100%	90%	0.090
Five-year EFS	60%	37.6%	0.080
Five-year NRM	0	10%	0.080
Five-year GF	18%	10%	0.500
Five-year CIR	36.3%	50%,	0.200
Median follow-up	102 months (95% CI, 90.6–114.3)		

*PNH* paroxysmal nocturnal hemoglobinuria, *IST* immunosuppressive therapy, *HSCT* hematopoietic stem cell transplantation, *OS* overall survival, *EFS* event-free survival, *CIR* cumulative incidence of relapse, *GF* graft failure, *NRM* non-relapse mortality, *CI* confidence interval

## Discussion

PNH clones are frequently observed in patients with AA, reflecting a unique intersection between immune-mediated bone marrow failure and clonal hematopoiesis. In our study, the presence of a PNH clone at diagnosis was independently associated with improved response to IST, reduced relapse, and superior OS. These findings corroborate a growing body of literature highlighting the favorable prognostic implications of PNH clones in AA.

Mechanistically, PNH clones arise due to somatic mutations in the PIGA gene and lack GPI-anchored proteins, rendering them less susceptible to immune-mediated destruction. Wong et al. proposed that these GPI-deficient cells evade cytotoxic T-cell–mediated attack, allowing selective survival within the autoimmune marrow failure milieu. Transcriptomic profiling further supports this immune escape hypothesis, suggesting a “silent” immunologic phenotype that correlates with a better response to IST and a lower risk of GVHD after HSCT [7].

Multiple studies have affirmed this favorable response pattern. Shilova et al. reported that 58% of patients with AA harbored a PNH clone, which was often stable over time, with larger clones (> 25%) more likely to be associated with hemolysis. Their study emphasized that small clones (< 10%) were clinically silent yet common, supporting our observation that clone size alone should not dictate therapeutic urgency but warrants longitudinal monitoring [6]. Similarly, Wang et al. demonstrated significantly higher response rates to IST (68–70%) among PNH-positive patients with SAA, coupled with faster hematologic recovery and more rapid immune modulation [5].

Scheinberg et al. and Ren et al. further refined this understanding by showing that small or moderate PNH clones, particularly when detected via FLAER, predicted early response to IST. In Scheinberg’s cohort, post-IST clone expansion was rare, and clinical complications occurred only when clone sizes exceeded 50% [8, 9]. Ren et al. emphasized the prognostic utility of FLAER in early-phase IST response, suggesting that even minute clones confer a survival advantage and lower early mortality [9].

Meta-analyses provide robust validation of these findings. Tu et al. and Wang et al. demonstrated that the presence of a pretreatment PNH clone was associated with significantly improved 6- and 12-month response rates, with Tu et al. reporting an odds ratio (OR) of 2.85 and Wang et al. reporting an OR of 2.73 [10, 11]. Li et al. further reported that clone positivity was associated with a higher risk of developing clinical PNH over time, suggesting that although favorable for IST response, clonal expansion may carry long-term risks of transformation [12].

Fattizzo et al., in a large retrospective cohort study, confirmed these trends. Among patients with AA, the presence of a PNH clone significantly improved response rates to both IST (78% vs. 50%) and HSCT (97% vs. 77%). Importantly, even clones as small as 0.01% retained prognostic relevance, reinforcing the need for high-sensitivity screening at diagnosis [13]. These results align with the survival outcomes observed in our cohort, in which clone positivity predicted both short-term therapeutic benefit and long-term survival.

Kulagin et al. further highlighted the predictive value of PNH clones in a prospective study of 125 patients with AA. PNH-positive patients demonstrated higher response rates to both first- and second-line IST, with 42% achieving complete remission compared with 16% of PNH-negative patients [14]. The study identified both clone presence and absolute reticulocyte count as independent predictors of favorable outcomes. These findings are echoed in our data, in which PNH clones frequently co-occurred with markers of preserved hematopoietic reserve.

The role of PNH clones extends beyond IST to HSCT. In our HSCT subgroup, PNH-positive patients achieved excellent OS and a significantly lower incidence of chronic GVHD, supporting a possible protective effect of GPI deficiency against alloreactive T-cell–mediated injury. Similar findings were reported by Yılmaz et al., who found no difference in OS or graft failure–free survival between patients with classical PNH and those with PNH-associated AA undergoing allogeneic hematopoietic cell transplantation, although they did not specifically evaluate GVHD outcomes according to clone status [15].

This observation is reinforced by Lee et al., who reported a 5-year OS of 87.9% among 33 transplanted patients with PNH or PNH-associated AA, with reduced-intensity conditioning (RIC) regimens effectively clearing clones within 2 months [16]. Liu et al. noted faster engraftment in patients with classical PNH compared with those with PNH-associated AA, but similar OS and GVHD outcomes overall [17]. Markiewicz et al. demonstrated complete clone clearance in more than 80% of patients and identified pretransplant hemolysis as a major determinant of OS, an important consideration in clone-driven risk stratification [18].

Gavriilaki et al. conducted two retrospective studies highlighting the complex prognostic role of PNH clones in AA. In a cohort of 24 patients with SAA undergoing allogeneic transplantation, the presence of large PNH clones (≥ 60%) was associated with fatal thrombotic complications and lower 10-year survival, suggesting a thromboinflammatory synergy exacerbated by GVHD and the absence of complement inhibition [19]. In a separate dual-center study, PNH clones were more prevalent among nontransplanted patients with AA, in whom they were linked to favorable responses to IST. However, in the transplanted subset, PNH clone presence independently predicted worse survival and a higher risk of fatal transplant-associated thrombotic microangiopathy [20]. These studies underscore the contrasting impact of PNH clones, favorably influencing IST outcomes while conferring higher transplant-related risks.

Eculizumab has emerged as a promising adjunct in this setting. Cooper et al., DeZern et al., and Vallet et al. all reported encouraging survival and GVHD outcomes when eculizumab was administered before or during transplantation [21–23]. Notably, DeZern et al.’s cohort achieved 100% survival, with no GVHD or relapse reported [22]. However, long-term data remain limited. Mei et al. reported two relapses and three deaths among eight post-HSCT recipients of eculizumab, raising questions regarding clonal evolution and disease control in the posttransplant period [24].

Finally, Ussowicz et al. comprehensively reviewed the current role of HSCT in PNH and PNH-associated AA, emphasizing that clone eradication, improved OS, and mitigation of GVHD are achievable with modern RIC protocols [25]. Our findings align closely with these data, suggesting that PNH-positive patients, particularly those with marrow failure phenotypes, may derive dual benefit from IST and HSCT. Individualized strategies, including peri-transplant eculizumab, may further optimize outcomes.

In conclusion, the presence of a PNH clone in AA predicts favorable outcomes with IST, including improved OS and lower relapse rates. Following HSCT, PNH-positive patients achieved excellent survival with a reduced incidence of chronic GVHD. These findings underscore the prognostic—and possibly protective—role of PNH clones and warrant prospective evaluation of clone-guided treatment algorithms in AA.

## Data Availability

All are available upon reasonable request from correspondence author.
